# Overcoming the Shadow of Expertise: How Humility and Learning Goal Orientation Help Knowledge Leaders Become More Flexible

**DOI:** 10.3389/fpsyg.2019.02505

**Published:** 2019-11-07

**Authors:** Mai P. Trinh

**Affiliations:** Faculty of Leadership and Interdisciplinary Studies, College of Integrative Sciences and Arts, Arizona State University, Tempe, AZ, United States

**Keywords:** expertise, flexibility, humility, learning goal orientation, performance goal orientation

## Abstract

Although experts are valuable assets to organizations, they suffer from the curse of knowledge and cognitive entrenchment, which prevents them from being able to adapt to changing situational demands. In this study, I propose that experts’ performance goal orientation resulting from pressures to perform contributes to their flexibility, but this mechanism can be moderated by learning goal orientation and humility. Data from a small sample of healthcare professionals suggested that performance goal orientation partially explained the mechanism of why experts may be inflexible. Humility, both as self-report and other-report measures, was found to be the most consistent moderator of this indirect effect. Experts with low levels of humility suffered from the negative effects of performance goal orientation, leading them to be less flexible compared to their counterparts with higher levels of humility. Experts who reported high levels of humility, on the other hand, were perceived to be more flexible as their expertise increased. Meanwhile, learning goal orientation partially moderated the indirect effect of expertise on flexibility through performance goal orientation. These findings lead to new conversations on how to get experts unstuck and highlight the importance of developing humility as both a personal virtue and a strategic advantage for organizations.

## Introduction

In September 2013, Nokia—once one of the most valuable companies on earth—sold its handset business to Microsoft for $7.2 billion, only a fraction of its past worth. The rapid downfall of the tech giant was largely attributed to its inability to respond to disruptive innovations, specifically the appearance of the Apple iPhone in 2007. During the press conference announcing Nokia being sold to Microsoft, Nokia’s CEO famously said while tearing up: “We didn’t do anything wrong, but somehow, we lost” (Jawabra, 2015).

Nokia did not do anything wrong; it was expert in what it was doing. It only failed to catch up with change. In today’s world where Heraclitus’s famous saying “the only constant in life is change” has never been truer, acquiring and training experts to be able to quickly respond to the changing environments proves to be a major challenge. While knowledge experts tend to perform, make decisions, and solve problems better than novices (Ericsson and Charness, 1994; Sonnentag and Kleine, 2000; Dreyfus and Dreyfus, 2005; Kahneman and Klein, 2009; Salas et al., 2010), research has shown that they are slower in adapting to change. Experts suffer from the “curse of expertise” (Camerer et al., 1989; Hinds, 1999), making them unable to unlearn things they already know even when the situation demands it. They are slow to respond to situational changes, such as when instructions change (Marchant et al., 1991) or when their problem-solving strategies are severely affected by external conditions (Canas et al., 2003).

I set out to seek explanations for experts’ inflexibility and what can be done to help them overcome this problem. Despite abundant evidence about experts’ lack of flexibility compared to novices, no tangible solution has been found. From a macro perspective, Hinds and Pfeffer (2003) suggested that organizations could try using people with intermediate level of knowledge instead of experts, as well as implementing organizational practices aiming to promote and reward knowledge sharing between experts and novices. While these recommendations could potentially generate organizational level impact, not using experts in organizations equals forgoing the advantages that experts bring, which may be counterproductive to the organization’s success (Littlepage and Mueller, 1997; Bunderson, 2003; Baumann and Bonner, 2004). From a cognitive standpoint, prior research argues that experts’ inflexibility is due to cognitive biases and rigidity resulting from their own training (Bilalić et al., 2007; Dane, 2010). Nevertheless, there has been no empirical evidence to date testing these explanations in the organizational context. Furthermore, while they can explain why experts are less flexible compared to novices, they do not explain why some experts may be more flexible than others.

To address this research gap, this study draws upon Dweck’s (1986; 1999) implicit theories of abilities to examine how one’s belief about one’s own ability may affect their flexibility. This theory suggests that *entity theorists* tend to have a *fixed mindset* because they do not believe that their ability can change through practice and learning, and thus adopt a *performance goal orientation* (PGO), seeking recognition by performing well. On the other hand, *incremental theorists* tend to have a *growth mindset* because they believe that they can learn and improve their ability, thus they adopt a *learning goal orientation* (LGO), focusing on developing their skills and seeking developmental feedback. I argue that today’s knowledge experts are under a great deal of pressure to maintain their superior performance, credibility, and reputation, which makes them prone to adopt a PGO. This PGO in turn makes them risk-averse, afraid to make mistakes, and likely to miss out on opportunities to learn or try different approaches, hence in flexible (Elliott and Dweck, 1988). On the contrary, LGO would help loosen experts’ performance mindset and help them become more flexible. The ability to overcome this performance mindset and the overconfidence trap often associated with expertise will also depend on how accurately one views one’s abilities and limitations—namely, one’s humility (Bauer and Wayment, 2008). Humility facilitates learning and development by helping people be open to new paradigms, acknowledge their own limitations and mistakes, accept failures as-is, be able to ask for advice, develop others, and perform better (Vera and Rodriguez-Lopez, 2004). Altogether, I propose that experts with a strong LGO and/or a high level of humility will overcome pressures to perform and tend to be more flexible than their counterparts without these virtues.

This study contributes to the literature in two important ways. First, by testing PGO as a mediating variable in the expertise-inflexibility relationship, this study extends previous research that has primarily examined why experts are less flexible than novices and offers one of the first pieces of empirical evidence explaining why some experts may be more flexible than others. Understanding the mediator of this relationship provides theoretical insights into the mechanism through which expertise affects flexibility. Second, by examining the moderating effects of LGO and humility on this same relationship, this study is among the first to suggest empirical interventions to help experts become more flexible. Not only will this insight advance socio-cognitive theories of expertise acquisition and training, but it will also have practical implications for companies and organizations to provide professional development opportunities to their skilled workforce.

## Literature Review and Hypotheses

Expert: “A person that has made every possible mistake within his or her field.”
∼ *Niels Bohr (1885–1962), Danish scientist and Nobel laureate*.

To better understand expert performance, it is helpful to first consider the concept of expertise and how it has been defined in the extant literature. In this section, I first review the use of expertise in psychological and organizational sciences, then present evidence of pitfalls in expert performance, namely the lack of flexibility in changing situations.

### Conceptualizing Expertise

In the history of expertise research, scholars have taken two main approaches to studying expertise. The first, called the relative approach (Chi, 2006), compares the performance of experts and novices in terms of basic cognitive processes such as memory and categorization. This approach flourished after the classic study of de Groot (1946) in which expert chess players were found to perform well above beginners in terms of the ability to reconstruct midgame boards that they had seen for only five seconds. In this approach, expertise is defined relatively to novice in a continuum, with the assumption that it is something that can be acquired. The goal of studying relative expertise is to gain understanding as to which cognitive skills are present in experts and not novices in order to train less experienced people to acquire those skills (Chi, 2006).

Another group of researchers, most notably Ericsson and associates, takes a different approach called the expert performance approach (Ericsson and Ward, 2007) or the absolute approach (Chi, 2006). Instead of studying basic cognitive processes, they concentrated on the behavioral aspect of expertise and tried to understand the mechanisms underlying consistent superior performance in order to draw implications for training and interventions. Expertise is defined as a high level of domain-specific knowledge and skills acquired through experience and practice (Chi et al., 1982; Dreyfus and Dreyfus, 1986; Ericsson and Charness, 1994; Feltovich et al., 2006). The study of expert performance in this approach is captured by three stages: (1) identify the environment in which experts excel and develop tasks representative of this environment, (2) assess the underlying mechanisms that account for excellent performance in these representative tasks, and (3) examine how these mechanisms affect and are affected by experience, learning, and practice, in order to develop implications for effective coaching (Williams and Ericsson, 2008). Studies using this approach have revealed that the acquisition of expertise is gradual and takes at least 10 years of intense preparation and deliberate practice (Ericsson et al., 1993; Ericsson and Charness, 1994; Ericsson, 2004; Ericsson and Ward, 2007).

These two approaches differ not only in the way expertise is defined but also in the domains of expertise they study. The first approach often studies knowledge experts in the lab, such as chess players, medical doctors, financial analysts, or tax accountants—those whose performance largely depend on their general mental ability and cognitive skills. Meanwhile, the second approach focuses more on experts in sports, music, and performing arts, in which physical and/or aesthetic ability is also required as proof of expertise. Criteria of extraordinary performance are also more clearly defined with this second population, as winning or losing is often the direct evidence of performance. While both approaches provide valuable insights into the superior skills of experts, the former is more applicable in organizations where performance is evaluated in terms of intellectual and not physical or artistic outcomes. Because knowledge experts’ problem-solving and decision-making are the foci of this study, I adopt Asare and Wright’s definition of expertise as “knowledge in a particular domain, including the ability to identify and evaluate relevant evidence, recognize patterns, consider transaction and opportunity costs, and properly represent a decision problem” (Asare and Wright, 1995, p. 172).

### Experts’ Lack of Flexibility

Experts’ inflexibility within their domain of expertise is well documented as a limitation preventing their effectiveness and consistent superior performance (Dane, 2010). Flexibility is loosely defined as one’s ability to adapt to changes, adjust to new circumstances, and update one’s own knowledge and skills to meet situational demands. During and after the process of acquiring expertise, many experts develop habitual responses (Wood and Neal, 2007) and have difficulty changing their behaviors (Betsch et al., 2001) even when such responses become incompatible with the new situation (Bagozzi and Dholakia, 2005). Camerer et al. (1989) coined the term “the curse of knowledge” to describe how experts were inclined to keep gathering irrelevant information despite their best interest to ignore this irrelevant information. Experts were unable to forget what they already knew, and falsely recalled more information than provided in a lab experiment (Castel et al., 2007). Similarly, Marchant et al. (1991) reported in a series of three experiments studying introductory tax students and experienced tax practitioners that when new rules were introduced, they interfered with experts’ reasoning and reduced experts’ performance, while students were able to learn quickly and their performance improved.

From the relative approach’s point of view, two cognitive explanations have emerged to explain why experts tend to be less flexible than novices. In a series of lab experiments having people solve chess puzzles, Bilalic and colleagues demonstrated that expert chess players were prone to the Einstellung effect, which occurs when the appearance of the first solution coming to mind prevents a better solution from being found (Bilalić et al., 2008). The authors observed that even though the expert players reported that they were looking for a better solution after finding the first one, their eye movements showed that they continued looking at features of the problem related to the solutions they had already thought of. The presence of the first, non-ideal solution reduced experts’ problem solving ability by three standard deviations of skill levels (Bilalić et al., 2007). This behavior is similar to the confirmation bias in psychology (Tversky and Kahneman, 1974; Nickerson, 1998; Jonas et al., 2001): once experts hold a certain opinion about something, they will tap into their vast expert knowledge to find evidence to defend their opinion (Mercier, 2011).

The second explanation came from Dane’s (2010) cognitive entrenchment framework about the trade-off between expertise and flexibility. He looked at the cognitive structure of expert knowledge and suggested that as novices learned to become experts, their cognitive schemas became larger, more complex, more interrelated, more detailed, and more accurate. Reinforced over time by the continual repeated practice and application, these schemas also tended to be more stable, thus leading to experts being “cognitively entrenched” or unable to move beyond their specific domain schemas. As someone becomes an expert, (s)he is already fixated on the “best” way to problem-solve and is not likely to change his/her way of doing things. Dane also proposed that there were two possible solutions to help experts become more flexible: being engaged in a dynamic environment within their domain, or focusing more on outside-of-domain tasks (Dane, 2010). Unfortunately, no study to date has empirically tested these two propositions, nor quantified cognitive entrenchment in organizations.

While both Dane’s and Bilalic’s explanations suggest reasons why experts are less flexible than novices, they do not explain why some experts may be more or less flexible than others. The cognitive mechanisms revealed in their theoretical frameworks would suggest that experts at the same level in the same domain would be cognitively biased or entrenched in the same manner, which is not what we observe in reality (Dreyfus and Dreyfus, 2005). Furthermore, Bilalic observed that the greater the level of expertise, the less chess experts were susceptible to the Einstellung effect (2007), but it was unclear why that happened. Since the relative approach offers little to answer my research question, I look further into research using the absolute approach for an alternative explanation.

Using the absolute approach, Ericsson (2004) argued that experts’ inflexibility was the result of a lack of continuous deliberate practice. Deliberate practice distinguishes professionals who reach a stable performance plateau within a short period of time and expert performers who keep improving their performance for years. He explained this distinction in terms of automaticity:

As individuals adapt to a domain and their performance skills become automated, they are able to execute these skills smoothly and without apparent effort. As a consequence of automation, performers lose conscious control over execution of those skills, making intentional modifications difficult. Once the automated phase of learning has been attained, performance reaches a stable plateau with no further improvements, which is consistent with [Sir Francis] Galton’s assumption of a performance limit… The key challenge for aspiring expert performers is to avoid the arrested development associated with automaticity and to acquire cognitive skills to support their continued learning and improvement. The expert performer counteracts the tendencies toward automaticity by actively acquiring and refining cognitive mechanisms to support continued learning and improvement (Ericsson, 2004, p. S70/S73).

In other words, in order to continue learning, adapting, and improving their performance, experts need to constantly challenge themselves to change, acquire performance feedback, and refine their skills. All of these prove difficult to sustain over time. Research has shown that reduced regular practice is the primary reason expert performance declines (Ericsson, 2004; Krampe and Charness, 2006), while the lack of feedback or willingness to seek feedback inflates experts’ confidence and reduces their judgment accuracy (Oskamp, 1965; Sniezek and Van Swol, 2001; Fischer and Budescu, 2005; McKenzie et al., 2008). The limitations of this explanation are that it was drawn mostly from experts in competitive fields such as sports and music, and that empirical evidences of the causes (for example, how much an expert changes from performance feedback) have been surprisingly scarce (Williams and Ericsson, 2008). This explanation also focuses mainly on the behavioral aspect of expertise, which is difficult to observe and measure in organizational settings.

In my quest to explain why some experts in organizations are more flexible than others and find solutions to help experts become more flexible, I move beyond the cognitive and behavioral realms to explore motivational factors affecting experts’ performance and their ability to adapt. Though not directly explaining why, a great deal of research has suggested that experts’ inflexibility is not a result of their inability to adapt, but rather a lack of willingness to absorb new information and change. When helping or teaching novices, experts fail to adjust their explanations to the novices’ level of understanding (Hinds et al., 2001), leading to novices having to ask for additional information not addressed in experts’ explanations (Wittwer et al., 2008). When working with other experts, they do not listen to advice (Yaniv and Kleinberger, 2000; Tost et al., 2012), ostracize others with different expertise (Jones and Kelly, 2013), and perform worse if too many experts are together in a group (Ashton, 1986; Groysberg et al., 2010). When communicating with managers on key issues, they may ignore managerial commands (Kellogg, 2009), refuse to be supervised (Alvesson, 2004), and cannot explain their expert insights in lay terms for managers to understand (Eppler, 2007). In the next section, I use a goal orientation framework to explain why organizational experts may often be motivated to be inflexible.

### A Goal Orientation Framework of Expert Inflexibility

The mechanisms leading to experts’ inflexibility could be explained using Dweck’s goal orientation framework associated with her implicit theories of abilities (Dweck, 1986, 1999; Dweck and Leggett, 1988). Entity theorists believe that intelligence is fixed and tend to hold a PGO, while incremental theorists believe that intelligence is malleable and tend to hold a LGO. A PGO propels individuals to demonstrate their competence via task performance, while a LGO makes them focus on continuous learning and development. Depending on which orientation is stronger, people respond differently to learning opportunities. Learning goal oriented people tend to see advice and feedback as useful in helping them improve performance and task mastery; while performance goal oriented people view feedback as an often derogatory evaluation of their competency (VandeWalle, 2003). Individuals with a strong LGO seek challenges that foster learning and persist in order to learn and improve their competence, while performance goal oriented individuals try to avoid failure and any display of incompetence (Dweck and Leggett, 1988).

Later empirical studies in organizations have revealed that PGO and LGO are not two ends of a spectrum but instead two independent constructs (Button et al., 1996; Ford et al., 1998). In other words, individuals may have both a high PGO, striving to prove their competence to others, seeking favorable judgments and avoiding negative judgments, and at the same time have a high LGO, aspiring to develop competence by acquiring and mastering new skills. Dweck’s work has found that in the face of challenges, PGO may contribute debilitating factors such as loss of efficacy, defensive withdrawal of effort, attention division between goal and task, and negative affect (Dweck and Leggett, 1988). Meanwhile, LGO contribute facilitating factors such as continued belief in efficacy or effort, undivided attention, affect being channeled into tasks, and continuous intrinsic rewards for trying to meet challenges (Dweck and Leggett, 1988).

Facing pressure to perform, experts can hardly afford to fail or to make mistakes. Shanteau asserted that “to be accepted as an expert, it [was] necessary to act like one” (Shanteau, 1992, p. 257). People are more likely to listen to not only those who are experienced and knowledgeable (Yaniv and Kleinberger, 2000; Yaniv, 2004; Soll and Larrick, 2009), but also those who express confidence in their advice to others (Sniezek and Van Swol, 2001; Van Swol and Sniezek, 2005; Soll and Larrick, 2009). This social perception creates pressure for experts to present themselves as confident in their judgments and decisions (Littlepage and Mueller, 1997; Bonner and Bolinger, 2013) and to be consistent in what they say and do, because inconsistency is often perceived as incompetent or irrational (Dessalles, 2007; Kurzban and Aktipis, 2007; Mercier, 2011). In fact, it is much more difficult for an expert to gain reputation than to lose it (Yaniv and Kleinberger, 2000; Sniezek and Van Swol, 2001; Tinsley et al., 2002). All of these social pressures force experts to create a professional image of themselves—one that is all-knowing, confident, never making any mistake or changes (Yanow, 2009; Sperber et al., 2010). As a consequence of trying to protect their credibility, many experts fall victim to defensive mechanisms (Argyris, 1985, 1994) and become reluctant to seek feedback or knowledge from others. Leonard and Sensiper (1998) described cases in which nurses were hesitant to suggest patient treatments to physicians who were of higher status, even though nurses may have good ideas based on their intensive experience and direct care of the patients. Edmondson et al. (2000) reported similar situations in operating rooms, where nurses and other low-status members of the operating team hesitated to share their expertise with surgeons because surgeons responded negatively to advice from them. All of these previous empirical results suggest that experts are prone to adopt a PGO to prove their status and protect their credibility, and this focus on performance in turn prevents them from being able to learn and change.

Hypothesis 1: Performance goal orientation mediates the relationship between expertise and flexibility.

Although experts are under pressure to perform, the degree to which this performance-oriented environment affects how flexible they are varies. Dweck’s work has shown that PGO is a maladaptive pattern of behavior—meaning that when facing failures and setback, strongly performance-oriented people tend to blame their intellectual ability (Henderson and Dweck, 1990), feel helpless (Dweck and Leggett, 1988), and lose confidence in performing future challenging tasks (Dweck et al., 1995). They have a difficult time adapting their behavior to sustain future success and consequently display patterns of decreasing performance (Henderson and Dweck, 1990; Zhao and Dweck, 1994). Argyris (1986, 1991) observed a similar phenomenon: when organization consultants—very intelligent and skilled professionals with MBA degrees from the top three or four U.S. business schools—encountered failures or setbacks, they reacted defensively and blamed the clients for being uncooperative, arrogant, and not helpable. Argyris argued that this defensive reasoning resulted from the fear of failure in this demanding context prevented these consultants from learning, changing, and actually improving their performance (Argyris, 1991). In fact, performance-oriented people are always concerned with how their work is evaluated or who will evaluate their work (Dweck, 1986, 1999; Button et al., 1996). If nothing intervenes, they will continue to sink deeper into this maladaptive patterns of behavior that Argyris termed the “doom loop” (Argyris, 1991, p. 7).

It is important to emphasize again that LGO and PGO are not opposite sides of a spectrum and that strong LGO and strong PGO can co-exist in the same person (Button et al., 1996). With such people, both priorities—performing well and developing skills—would be ranked equally high, hence they would dedicate time to learn and improve in addition to meeting performance benchmarks in their jobs. How much they can learn and adapt will mitigate the negative effect that their expertise brings to their flexibility. This is possible because the adaptive pattern of behaviors associated with LGO has been confirmed in many empirical studies and found to be positive predictor of college GPA, self-esteem (Button et al., 1996), feedback seeking behavior (VandeWalle and Cummings, 1997; Anseel et al., 2015), and knowledge and performance (Bell and Kozlowski, 2002). When learning-oriented people fail a task, they are likely to identify factors that may have mediated the negative outcomes such as lack of effort or wrong strategies (Henderson and Dweck, 1990; Dweck et al., 1995), and thus able to use this reasoning to improve subsequent performance (Henderson and Dweck, 1990). They are also less likely to abandon a task than purely performance-oriented people (Button et al., 1996).

Since previous research has shown that motivation to learn leads to actual learning (Colquitt and Simmering, 1998), people who seek to improve their skills would be more motivated to try different approaches to learning and doing things, hence being able to apply new learnings and adapt quickly should situations demand so. Thompson (1999) discovered that professionals who were more self-directed in their learning behaviors had higher adaptive flexibility than those who were not. Moreover, learning-oriented experts will be more likely to see others’ advice and feedback as opportunities for growth and development instead of as threats to their status and reputation. VandeWalle and Cummings (1997) reported that people with LGO perceived feedback from others as more valuable and less costly, thus they engaged in more feedback seeking behaviors. A recent meta-analysis by Anseel et al. (2015) reveals a positive relationship between LGO and feedback seeking behaviors, suggesting that people who want to learn and develop themselves give greater weight to the value of feedback over the self-representation cost associated with feedback (e.g., negative image, being seen as inferior).

Hypothesis 2: The indirect effect of expertise on flexibility will be conditional on LGO such that this effect will be more negative among those with lower LGO than among those with higher LGO.

### Humility as a Virtue to Improve Expert Flexibility

A major issue with experts’ inflexibility that is not directly explained by the goal orientation framework is their rigidity in opinion and overconfidence. Research has shown that subjective experience of power could inflate one’s perception of personal control (Fast et al., 2009) and increase one’s confidence in their own judgments and opinions (Brinol et al., 2007; See et al., 2011). Experts engage in egocentric advice discounting, overweighting their own opinions and underweighting others’ (Yaniv and Kleinberger, 2000; Krueger, 2003; Yaniv, 2004). Even when comparing their judgment with chance events judged to be equally likely, experts still favor their own expert knowledge over the uncertainty (Heath and Tversky, 1991). Consequently, it leads them to resist being dependent on others (Galinsky et al., 2008) and to refuse to listen to others’ input (Tost et al., 2012), therefore reducing their judgment accuracy (See et al., 2011). These recent findings are consistent with previous research about how (high) power and status are among the most detrimental factors preventing new learning in groups and organizations (Brooks, 1994; Edmondson, 2002).

Experts’ sense of overconfidence is not likely to be resolved by having a strong LGO, but instead by developing a sense of humility. Long viewed as a linchpin of wisdom in Eastern philosophical traditions, humility has received increasing attention from organization scientists as a virtue in positive organizational scholarship (Cameron et al., 2003), a desirable characteristics of great leaders (Collins, 2001; Owens et al., 2013), and a cornerstone of organizational learning (Vera and Rodriguez-Lopez, 2004). Humility, together with honesty, recently emerged as the sixth factor of the Big-Five personality traits in social psychology (Ashton and Lee, 2005). Researchers generally agree that humility is a multi-dimensional, adaptive strength (Templeton, 1997; Tangney, 2000) that reflects an accurate view of oneself and one’s limitations (Bauer and Wayment, 2008), an awareness that something is greater than the self, a sense of appreciation toward others, and openness to feedback (Owens et al., 2011, 2013; Ou et al., 2014).

Even though there has been no empirical study linking expertise and humility, some evidence exists about how one’s humility brings about positive organizational outcomes through improving one’s relationship with others. Personal humility coupled with a strong sense of professional resilience are what make leaders not just good, but great (Collins, 2001). Owens and Hekman (2012) revealed that humble leaders created positive relationships with their followers by being compassionate about followers’ developmental journey and validating followers’ feelings of uncertainty. By being humble, leaders facilitate team learning and increase employee engagement and job satisfaction (Owens et al., 2013). Humble CEOs also make followers feel empowered, which in turn increase their work engagement, affective commitment, and job performance (Ou et al., 2014). Vera and Rodriguez-Lopez (2004) posit that humble leaders benefit the firm because they possess such qualities as being open to new paradigms, being eager to learn from others, acknowledging their own limitations and mistakes and attempting to correct them, accepting failure with pragmatism, asking for advice, respecting others, and sharing honors and recognition with collaborators.

Furthermore, humility is an adaptive strength in the sense that it can help experts stay open to new possibilities and inquiries instead of insisting on advocacy and proving their own competence (Schein, 2013). Research has shown that when people feel that they have power, they tend to be overconfident about their decisions (Koehler, 1991; Fischer and Budescu, 2005; Brinol et al., 2007), which often leads to discounting others’ opinion (Yaniv and Kleinberger, 2000) and making bad decisions (Chatterjee and Hambrick, 2007; Moore and Healy, 2008; See et al., 2011; Hilbert, 2012). Because humility “requires a severe appraisal of oneself combined with a reasonably generous appraisal of others” (Newman, 1982, p. 283), it will reduce experts’ tendency to engage in egocentric discounting of advice. For experts, having humility means acknowledging that they do not have all the answers, that some other possibilities exist, and that someone else might have the better idea. Recognizing their shortcomings provides a call to broaden their perspectives (Ackerly, 2013), and to have a mental attitude and willingness to ask not only “How do I know?” but also “How would I know if I were wrong?” (Yanow, 2009). As Ed Schein asserted, “in an increasingly complex, interdependent, and culturally diverse world, we cannot hope to understand and work with people from different occupational, professional, and national cultures if we do not know how to ask questions and build relationships that are based on mutual respect and the recognition that others know things that we may need to know in order to get a job done” (Schein, 2013, p. 1–2). The pursuit of the “truth,” or their own continuing learning and development, would become more important to humble experts than their reputation. Therefore, having humility will reduce experts’ perceived social pressure to stay consistent and give them more liberty to try something new and change for the better (Vera and Rodriguez-Lopez, 2004). I expect that experts with higher levels of humility will be able to better withstand pressure to perform and therefore be more flexible than those with lower humility.

Hypothesis 3: The indirect effect of expertise on flexibility will be conditional on humility such that this effect will be more negative among those with lower humility than among those with higher humility.

**FIGURE 1:**
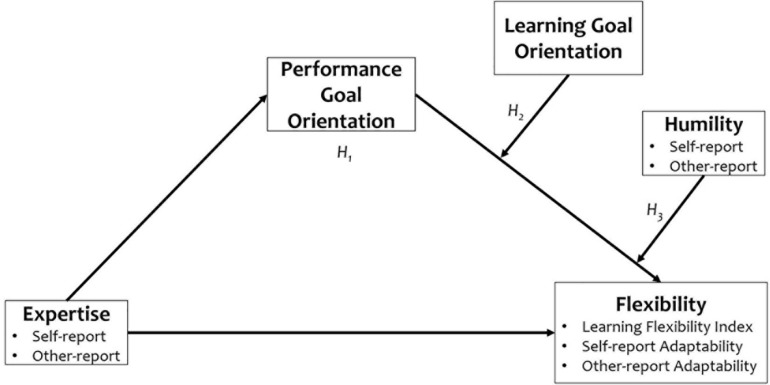
Conceptual model.

The conceptual model capturing all three hypotheses is presented in Figure 1.

## Materials and Methods

### Participants and Procedures

Participants in this study were employees of a large U. S. Midwestern health services organization. Participants were invited through email to complete a voluntary survey about adaptive performance at work. Participants responded to questions about their domain expertise, PGO, LGO, learning flexibility, humility, and adaptability. After they completed their self-report survey, they were asked to refer three to five colleagues who would be asked to fill out a rating form for them. The rating form for raters included other-report measures of expertise, expressed humility, and adaptability. The current sample includes 83 participants, 74% of whom were females. Average age was 47.74 years and average working experience was 18.82 years. Participants each received 0–5 colleague ratings, totaling 129 raters, and averaging 1.55 raters per participant.

### Measures

#### Control Variable

I controlled for participant’s sex because previous research has suggested that females tend to be more humble than males (Furnham et al., 2002; Owens et al., 2013). Furthermore, women receive less recognition for their achievements than men do and often deflect attention off of themselves (Fels, 2004). Therefore, if both a man and a woman have the same level of expertise, it is likely that the man will report higher expertise than the woman will, and he is also more likely to be perceived to have higher expertise than her. Controlling for sex effect will help keep these biases out of the results.

#### Domain Expertise

I used a five-item knowledge expertise scale taken from Johanna and Van der Heijden’s (2000) professional expertise scale (ranging from 1 = *Strongly disagree* to 7 = *Strongly agree*) to measure domain expertise and administered to both study participants and their raters. Johanna and Van der Heijden (2000) documented high reliability scores for this instrument (0.83 when self-rated by employees and 0.93 when rated by their supervisors). Moreover, they used Multitrait-Multimethod analysis to show that it was highly correlated with, yet distinctive from, related constructs such as meta-cognition, skills, social recognition, and growth and flexibility. Items for the self-report survey included “I have expert knowledge in my job domain,” “I consider myself competent to engage in in-depth discussions in the domain of my work,” “I consider myself competent to be of practical assistance to colleagues with questions in my areas of expertise,” “I am competent to handle the methods and materials in use in the domain of my work,” and “I am able to solve problems that occur at work at ease.” In the rater’s survey, raters were asked to rate their colleagues using the same statements, with the subject changed from “I” to “He/she.” Cronbach’s α for self-report expertise was 0.85 and for other-report expertise was 0.93.

#### PGO and LGO

PGO and LGO were each measured by the same eight items developed and validated by Button et al. (1996). In a series of four studies, these authors demonstrated that these instruments were reliable (α ranging from 0.68 to 0.81 for PGO and from 0.79 to 0.85 for LGO), had good factor structures, and that PGO and LGO were distinguishable. The items were anchored to a seven-point Likert scale (1 = *Strongly disagree*; 7 = *Strongly agree*). An example item for PGO was “I prefer to do things that I can do well rather than things that I do poorly.” An example item for LGO was “I prefer to work on tasks that force me to learn new things.” Cronbach’s α for PGO in the current sample was 0.85 and for LGO was 0.84.

#### Humility

In the self-report survey, humility was measured with seven semantic differential items on a scale from 0 to 100 with the following end-labels: humble/arrogant, modest/immodest, respectful/disrespectful, egotistical/not self-centered, conceited/not conceited, intolerant/tolerant, closed-minded/open-minded (Rowatt et al., 2006). The semantic differential measure was chosen because it was the best proxy for the arguably best available measure of self-report humility—the implicit association test (IAT) (Rowatt et al., 2006; Davis et al., 2010). The IAT could not be used because it required participants to be in a computer lab and to go through 240 trials while maintaining focus and attention. While the IAT was logistically challenging to implement, the semantic differential measure’s simplicity presented its advantage. Self-report humility measure was calculated as the average of participants’ response to these seven items.

In the rater survey, expressed humility was measured using nineteen items developed and validated by Ou et al. (2014) on a seven-point Likert scale (1 = *Strongly disagree*; 7 = *Strongly agree*). These 19 items were intended to measure six dimensions of humility: self-awareness (e.g., “My colleague actively seeks feedback, even if it is critical.”), appreciation of others (e.g., “My colleague takes notice of others’ strengths.”), self-improvement (e.g., “My colleague is willing to learn from others.”), low self-focus (e.g., “My colleague does not like to draw attention to himself/herself.”), self-transcendent pursuit (e.g., “My colleague devotes his/her time to the betterment of the society.”), and transcendent self-concept (e.g., “My colleague believes that no one in the world is perfect, and he/she is no better or worse than others.”). Other-report humility was calculated as the average of raters’ responses to these nineteen items. The composite reliability for the entire scale in this study was 0.93.

#### Flexibility

The dependent variable—flexibility—was operationalized in multiple ways in this study. When dealing with survey data, researchers are concerned that same-source and same-method ratings tend to be upwardly biased (Conway and Lance, 2010; Podsakoff et al., 2012). While the former concern can be partially alleviated by the use of multi-source ratings in this study, the latter is left unaddressed up until this point. Therefore, in addition to a Likert-scale measure of adaptability at work, I employed learning flexibility—the degree to which individuals alter their ways of approaching information and making decisions in different circumstances—as an additional measure of flexibility. As explained below, the forced-ranking format of learning flexibility qualified it to be a good different-method rating of flexibility.

##### Learning flexibility

Learning flexibility was measured by the Learning Flexibility Index (LFI) (Sharma and Kolb, 2011). Participants were presented with eight different learning contexts (e.g., “When I start something new”) and were asked to think of a specific example of each context in their life. They were then asked to rank four responses in terms of likelihood that they would use to respond to the situation. The four responses corresponded to four learning modes in Experiential Learning Theory (Kolb, 1984), namely concrete experience (CE), reflective observation (RO), abstract conceptualization (AC), and active experimentation (AE). For example, for the item “When I start something new,” the four responses are “I rely on my feelings to guide me” (CE), “I imagine different possibilities” (RO), “I analyze the situation” (AC), and “I try to be practical and realistic” (AE). If a participant ranked the four responses in this order (4-3-2-1), it meant that (s)he would most prefer relying on feelings or CE, followed by observations, followed by analysis, and least likely to be using experimentation. The LFI was defined as the degree to which respondents varied their preferred mode of response across the eight different situations and calculated as 1 – *W*, in which *W* was the Kendall’s coefficient of concordance. According to Sharma and Kolb (2011), with 8 learning situations and 4 learning modes, the mathematical formula for *W* is:

$$W=\frac{\begin{array}{c} 12\,\left(S\,u\,m_{C\,E}^{2}+S\,u\,m_{R\,O}^{2}+S\,u\,m_{A\,C}^{2}+S\,u\,m_{A\,E}^{2}\right)\\ -3\times 8^{2}\times 4\times {\left(4+1\right)}^{2} \end{array}}{8^{2}\,\left(4^{3}-4\right)}$$

Participant’s LFI score ranged from 0 to 1 with higher score indicating higher learning flexibility.

##### Adaptability

Adaptability was measured on a seven-point Likert scale (1 = *Strongly disagree*; 7 = *Strongly agree*) by 21 items adapted from Pulakos et al. (2000) taxonomy of adaptive performance. I presented a total of 54 items from the taxonomy to a panel of upper-level managers at the healthcare organization and asked them to select those which were desirable in their organization. The 21 items selected by this panel were included in both the self-report survey (α = 0.92) and the rater survey (α = 0.97). Sample items included “I think outside the given parameters to see if there is a more effective approach, “I effectively adjust plans to deal with changing situations,” and “I adjust to new work processes and procedures.”

### Analyses

#### Data Screening and Cleaning

Data was entered and screened in SPSS. The Little’s MCAR test was not significant (χ*^2^* = 187.50, *df* = 163, *p* > 0.05), suggesting that data was missing completely at random. Missing data was then replaced using expectation maximization procedure (Tabachnick and Fidell, 2013).

In order to calculate other-report scores of expertise, humility, and adaptability, I first calculated the interrater agreement *r*_*wg*_ for each participant’s rater scores in each of the three measures. I retained only responses with moderate agreement (*r*_*wg*_ > 0.50) (LeBreton and Senter, 2008) in the dataset, which meant different raters agreed with one another regarding a particular participant’s characteristic at a moderate level. Each participant’s score was calculated as the average rating of all raters. Participants with only one rater were excluded from the analysis because the single rating could not be triangulated with any other rating and could be potentially biased. The final dataset yielded 57 complete individual responses with ratings from 118 raters (2.07 raters per participant). Descriptive statistics and zero-order correlations are presented in Table 1. All variables are normally distributed and have sufficient variability.

**TABLE 1:** Descriptive statistics and zero-order correlations among study variables.

**Variable**	***M***	***SD***	**(1)**	**(2)**	**(3)**	**(4)**	**(5)**	**(6)**	**(7)**	**(8)**	**(9)**
1. Sex^1^	0.74	0.44	—								
2. Self-report expertise	6.38	0.56	–0.11	(0.85)							
3. Other-report expertise	6.57	0.50	0.03	0.10	(0.93)						
4. PGO	5.26	0.84	0.07	0.30^∗^	0.27^∗^	(0.85)					
5. LGO	6.26	0.52	0.23^†^	0.26^†^	–0.19	–0.02	(0.84)				
6. Self-report humility	75.78	11.11	–0.03	0.10	–0.14	0.05	0.08	—			
7. Other-report humility	6.03	0.58	0.22	–0.06	0.70^∗∗∗^	0.34^∗^	–0.03	0.00	(0.93)		
8. Self-report adaptability	5.97	0.53	0.16	0.36^∗∗^	–0.11	–0.14	0.55^∗∗∗^	0.41^∗∗^	–0.06	(0.92)	
9. Other-report adaptability	6.20	0.57	0.26^∗^	–0.11	0.71^∗∗∗^	0.28^∗^	0.04	0.01	0.91^∗∗∗^	0.06	(0.97)
10. Learning flexibility	0.68	0.16	0.20	−0.28^∗^	0.11	0.00	–0.12	−0.24^†^	0.17	–0.4	0.11

**N* = 57. Scale reliability α is presented in the diagonal of the matrix. ^1^Sex was coded as 0 = Male, 1 = Female. ^†^*p* < 0.10; ^∗^*p* < 0.05; ^∗∗^*p* < 0.01; ^∗∗∗^*p* < 0.001 (two-tailed).*

#### Hypothesis Testing

The four hypotheses were tested using the PROCESS Macro (Hayes, 2013) in SPSS 23. I first tested Hypothesis 1 using Model 4 (simple mediation) with 10,000 bootstrap samples to confirm the mediating effect of PGO on the relationship between expertise and flexibility.

I then used Model 14 with 10,000 bootstrap samples to test 18 separate conditional process models with two measures of expertise (self-report and other-report) as the independent variable, three measures of flexibility (LFI, self-report adaptability, and other-report adaptability) as dependent variables, and three moderators (LGO, self-report humility, and other-report humility). PGO was the mediator in all of these 18 models. Conditional process analyses were performed according to Hayes’s (2013) guidelines. PGO and the three moderators were mean-centered prior to analyses. Due to small sample size, I used 90% bootstrap confidence intervals for all mediation and conditional process analyses. A *post hoc* power analysis showed that with medium effect sizes and type I error probability α = 0.10, the conditional process analyses with 5 predictors yielded more than 0.99 power (1-β error probability) with a sample size of 57 (Murphy and Myors, 1999). In other words, despite the small sample size, the analyses presented here had sufficient statistical power to draw inferences with 90% confidence.

## Results

### Total Effects

Total effects reflect the influence of the independent variable(s) on the dependent variable in the absence of the mediator (Hayes, 2013). I first tested the total effects of expertise (self-report and other-report) on each measure of adaptability. Controlling for sex, self-report expertise had a negative effect on learning flexibility (*b* = −0.07, *p* < 0.05), while having a positive effect on self-report adaptability (*b* = 0.36, *p* < 0.01) (see Table 2). This suggested that people who reported to be high on expertise tended to report higher adaptability. However, when presented with different learning situations in the LFI, people with higher self-report expertise demonstrated less flexibility in responding to the eight different situations.

**TABLE 2:** Regression results showing total effects of expertise on flexibility.

**IVs**	**DV = LFI**	**DV = Self-report adaptability**	**DV = Other-report adaptability**	**DV = LFI**	**DV = Self-report adaptability**	**DV = Other-report adaptability**
Intercept	1.09	3.50	6.57	0.43	6.61	0.70
Sex	0.06 (0.05)	0.24 (0.15)	0.33(0.17)†	0.07 (0.05)	0.20 (0.16)	0.31(0.12)*
Self-report expertise	-0.07(0.04)*	0.36(0.12)**	−0.08(0.14)			
Other-report expertise				0.03 (0.04)	−0.12(0.14)	0.80(0.10)*⁣**
*R*^2^	0.11	0.17	0.08	0.05	0.04	0.56
*F* (df)	3.28(2,54)*	5.47(2,54)**	2.19(2,54)	1.45(2,54)	1.08(2,54)	34.50(2,54)*⁣**

**N* = 57. Effects are unstandardized regression coefficients. Standard errors are in parentheses. ^†^*p* < *0.10*; ^∗^*p* < 0.05; ^∗∗^*p* < 0.01; ^∗∗∗^*p* < 0.001 (two-tailed).*

Self-report expertise did not have any effect on other-report adaptability, and neither did other-report expertise have any effect on LFI and self-report adaptability. However, other-report expertise were strongly and positively related to other-report adaptability (*b* = 0.80, *p* < 0.001), suggesting that raters tended to associate expertise with adaptability when filling out the survey.

Altogether, these results suggested that there seemed to be common variance between measures of expertise and adaptability within same-source ratings. The LFI, being a force-ranking instrument instead of a Likert scale, helped control for this common method variance and may have been the more accurate indicator of flexibility. People who saw themselves as experts also thought of themselves as more adaptable, even though they demonstrated less flexibility when responding to different learning situations.

### Indirect Effects

Table 3 shows regression results testing the mediating effect of PGO on the relationship between expertise and flexibility. Significant indirect effects were observed in the paths from self-report expertise to self-report and other-report adaptability. These results suggested that when expertise was measured as self-report, PGO mediated the relationship between expertise and adaptability, both as self-report and as other-report measures. This finding partially supports Hypothesis 1. Controlling for sex, experts who reported to have a high level of expertise were likely to adopt a strong PGO, which in turn led to less self-report adaptability. This indirect effect (*b* = −0.09, *p* < 0.10) was small compare to the total effect. On the other hand, the strong PGO displayed by people with high self-report expertise led to higher adaptability perceived by their colleagues, making the indirect effect of self-report expertise on other-report adaptability through PGO a positive one (*b* = 0.10, *p* < 0.10). PGO did not mediate the effect of expertise on learning flexibility.

**TABLE 3:** Indirect effects of expertise on flexibility through the mediator PGO.

**IVs**	**DV = LFI**		**DV = Self-report adaptability**		**DV = Other-report adaptability**	
Self-report expertise	0.01 (0.01)		−0.09^†^ (0.06)		0.10^†^ (0.06)	
Other-report expertise		−0.00 (0.01)		−0.04 (0.04)		0.03 (0.04)
90% bootstrap LLCI	−0.01	−0.03	−0.22	−0.12	0.03	−0.02
90% bootstrap ULCI	0.03	0.02	−0.02	0.02	0.24	0.11

**N* = 57. Effects are unstandardized regression coefficients. Standard errors are in parentheses. LLCI, lower limit of confidence interval. ULCI, upper limit of confidence interval. An indirect effect is statistically significant if the 90% confidence interval does not contain zero. ^†^*p* < 0.10 (two-tailed).*

### Conditional Indirect Effects

Eighteen conditional process analyses were conducted to examine the indirect effects of two predictors (self-report and other-report expertise) on three outcomes (LFI, self-report adaptability, and other-report adaptability) through the mediator PGO, conditional on three moderators (LGO, self-report humility, and other-report humility). In the following section, I examine how these results support or do not support each hypothesis and explain the conditional indirect effects of each moderator using the accompanied graphs.

Analysis #03 examined the indirect effect of self-report expertise on self-report adaptability through the mediator PGO, conditional on the moderator LGO.

The statistical formulas for PGO and self-report adaptability are:

(1) $$P\,G\,O=i\,n\,t\,e\,r\,c\,e\,p\,t+a_{0}\times S\,e\,x+\,a_{1}\times E\,x\,p\,e\,r\,t\,i\,s\,e+e\,r\,r\,o\,r$$

(2) $$A\,d\,a\,p\,t\,a\,b\,i\,l\,i\,t\,y=i\,n\,t\,e\,r\,c\,e\,p\,t+\,b_{0}\times S\,e\,x+c^{\prime }\times E\,x\,p\,e\,r\,t\,i\,s\,e+b_{1}\times P\,G\,O+b_{2}\times L\,G\,O+b_{3}\times P\,G\,O\times \,L\,G\,O+e\,r\,r\,o\,r\times P\,G\,O\times \,L\,G\,O+e\,r\,r\,o\,r$$

The conditional effect of PGO on Adaptability in Formula (2) is *b*_1_ + *b*_3_ × *LGO*. The value of this effect depends on the value of the moderator LGO.

The indirect effect of Expertise on Adaptability through PGO conditional on LGO is therefore the product of the effect of Expertise on PGO [a_1_ in Formula (1)] and the conditional effect of PGO on Adaptability in Formula (2), making it

$$a_{1}\,\left(b_{1}+b_{3}\times \,L\,G\,O\right)$$

Table 4 presents the results of two regression analyses depicted in the two formulas (1) and (2) above. First, the mediator PGO was regressed on the control variable Sex and the IV Self-report expertise. Then the DV Self-report adaptability was regressed on the control variable Sex, the IV Self-report expertise, the mediator PGO, the moderator LGO, and the interaction term of the mediator and moderator PGO×LGO. As shown in Table 4, the interaction effect between PGO and LGO was marginally significant, indicating that the indirect effect of self-report expertise on self-report adaptability through PGO is indeed conditional on LGO, partially supporting Hypothesis 2.

**TABLE 4:** Results of conditional process analyses #03, 09, and 11.

**Variable**	**DV: PGO**	**DV: Self-report adaptability Moderator: LGO (Analysis #03)**	**DV: Self-report adaptability Moderator: Self-report humility (Analysis #09)**	**DV: Other-report adaptability Moderator: Self-report humility (Analysis #11)**
Intercept	−3.12(1.29)	3.81 (0.73)	2.42 (0.69)	6.63 (0.94)
Sex	0.19 (0.25)	0.11 (0.13)	0.29(0.12)*	0.29(0.16)*
Self-report expertise	0.47(0.20)*	0.33(0.11)**	0.52(0.11)*⁣**	−0.10(0.14)
PGO		-0.13(0.07)†	-0.22(0.07)**	0.20(0.09)*
LGO		0.43(0.12)*⁣**		
PGO × LGO		0.26(0.13)†		
Self-report humility			0.02(0.01)*⁣**	0.00 (0.01)
PGO × Humility			0.02(0.01)**	0.01(0.01)†
*R*^2^	0.10	0.45	0.50	0.22
*F (df)*	2.99(2,54)†	8.34(5,51)*⁣**	10.17(5,51)*⁣**	2.78(5,51)*

**N* = 57. Effects are unstandardized regression coefficient. Standard errors are in parentheses. ^†^*p* < 0.10; ^∗^*p* < 0.05; ^∗∗^*p* < 0.01; ^∗∗∗^*p* < 0.001 (two-tailed).*

**FIGURE 2:**
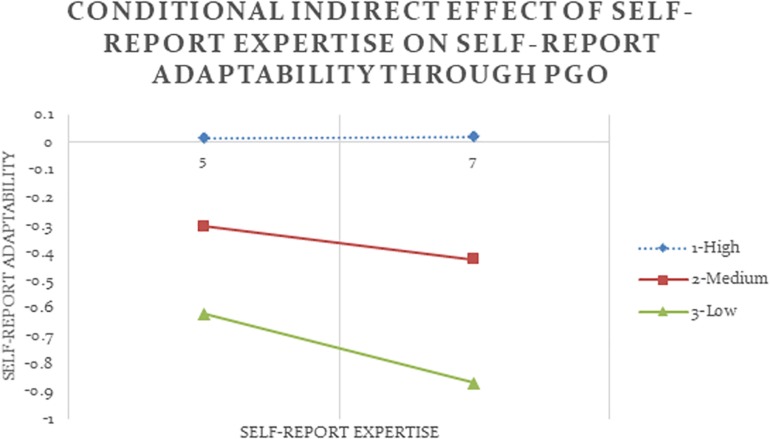
Conditional indirect effect of self-report expertise on self-report adaptability through PGO is significant at low and medium values of the moderator LGO with 90% confidence (Analysis #03).

To further examine this conditional indirect effect, I ran 10,000 times of bias-corrected bootstrap in SPSS. The conditional indirect effect was calculated at high, medium, and low values of LGO using Formula (3), with these values being 75th, 50th, and 25th percentiles of the moderator, respectively. Figure 2 illustrates the conditional indirect effect of self-report expertise on self-report adaptability through PGO at high, medium, and low values of LGO at high and low values of self-report expertise. The 90% bootstrap confidence intervals for the conditional indirect effects at medium and low values of LGO did not contain 0, indicating that these effects were statistically significant. These negative effects suggested that while all participants reported to be more adaptable as their expertise increased (*b* = 0.33, *p* < 0.05), only among experts who reported average or below average levels of LGO, higher self-report expertise led to stronger PGO (*b* = 0.47, *p* < 0.05), which then led to lower self-report adaptability (*b* = −0.13, *p* < 0.10). This conditional indirect effect was not significant among experts who reported high levels of LGO, denoted by the dotted line in Figure 2. LGO did not moderate any other set of relationship when expertise and flexibility were measured differently (Analyses #01-02, 04-06), thus Hypothesis 2 was only partially supported.

**FIGURE 3:**
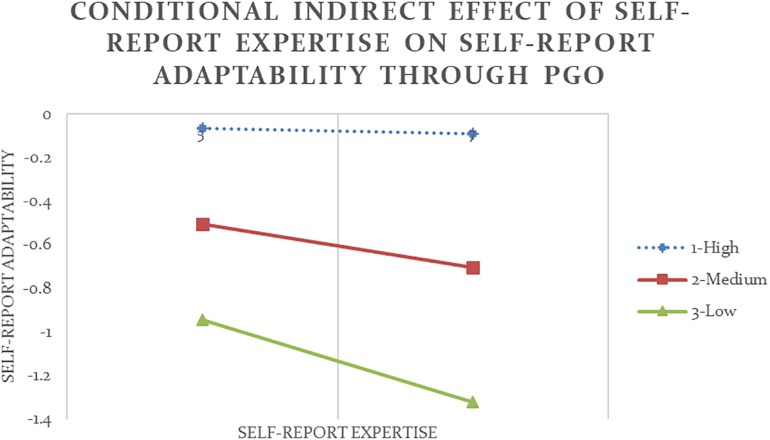
Conditional indirect effect of self-report expertise on self-report adaptability through PGO is significant at low and medium values of the moderator self-report humility with 90% confidence (Analysis #09).

Following the same procedure, self-report and other-report humility moderated the relationship between PGO and flexibility in five out of twelve occasions, partially supporting Hypothesis 3. As shown in Table 4 and Figure 3, the indirect effect of self-report expertise on self-report adaptability through PGO was negative and significant at low and medium values of the moderator self-report humility (Analysis #09). In other words, despite a strong positive direct effect of self-report expertise on self-report adaptability (*b* = 0.52, *p* < 0.001), only among people who reported to have an average or below average level of humility, higher expertise could lead to stronger PGO (*b* = 0.47, *p* < 0.05), but stronger PGO in turn led to lower adaptability (*b* = −0.22, *p* < 0.01).

**FIGURE 4:**
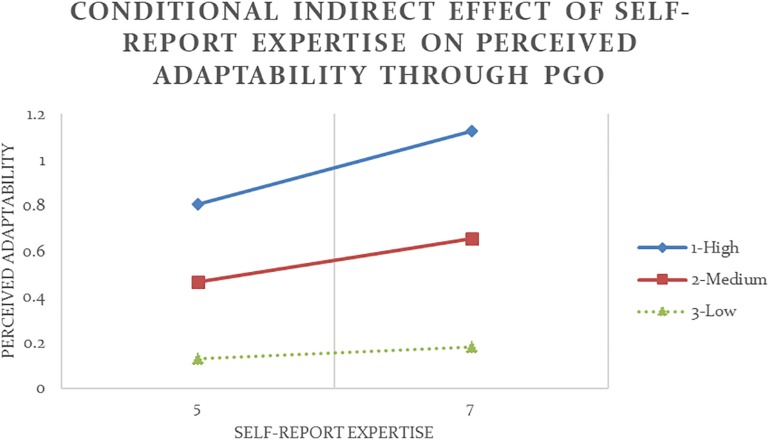
Conditional indirect effect of self-report expertise on other-report adaptability through PGO is significant at medium and high values of the moderator self-report humility with 90% confidence (Analysis #11).

**FIGURE 5:**
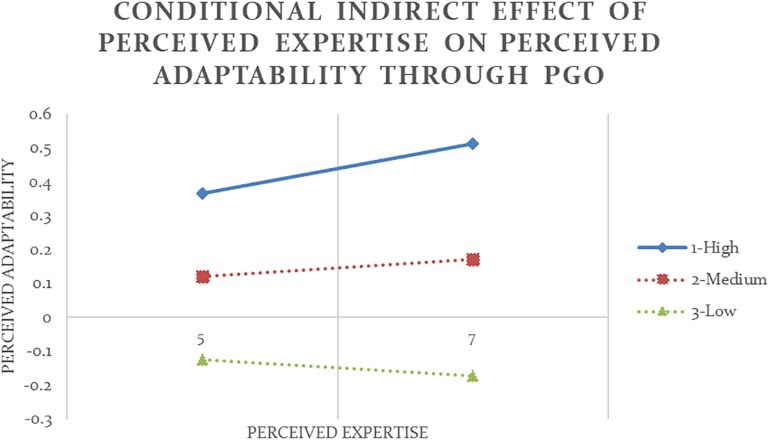
Conditional indirect effect of other-report expertise on other-report adaptability through PGO is significant at high values of the moderator self-report humility with 90% confidence (Analysis #12).

Analysis #11 (Table 4 and Figure 4) revealed that the indirect effect of self-report expertise on other-report adaptability through PGO was positive and significant at high and medium values of self-report humility. In other words, so long as they did not show too low level of humility, participants who reported to have high expertise also reported to have strong PGO (*b* = 0.47, *p* < 0.05) and subsequently were perceived to be more adaptable (*b* = 0.20, *p* < 0.05). Similarly, Analysis #12 (Table 5 and Figure 5) showed that although all participants tended to show more adaptability as their expertise increased (*b* = 0.77, *p* < 0.001), only among people who reported to have high level of humility was the indirect effect of other-report expertise on other-report adaptability through PGO also positive and significant.

**TABLE 5:** Results of conditional process analyses #12, 17, and 18.

**Variable**	**DV: PGO**	**DV: Other-report adaptability Moderator: Self-report humility (Analysis #12)**	**DV: PGO**	**DV: Other-report adaptability Moderator: Other-report humility (Analysis #17)**	**DV: Other-report adaptability Moderator: Self-report humility (Analysis #18)**
Intercept	−2.99(1.46)	0.92 (0.70)	−3.12(1.28)	6.39 (0.40)	4.94 (0.60)
Sex	0.11 (0.25)	0.30(0.11)*	0.19 (0.25)	0.10 (0.08)	0.13(0.07)†
Self-report expertise			0.47(0.20)*	−0.05(0.06)	
Other-report expertise	0.44(0.22)†	0.77(0.11)*⁣**			0.17(0.09)†
PGO		0.06 (0.06)		−0.00(0.04)	−0.02(0.04)
Self-report humility		0.01 (0.01)			
PGO × Self-report Humility		0.01(0.01)†			
Other-report humility				0.91(0.06)*⁣**	0.80(0.08)*⁣**
PGO × Other-report Humility				0.17(0.08)*	0.15(0.08)†
*R*^2^	0.07	0.61	0.10	0.84	0.85
*F* (df)	2.15(2,54)	15.90(5,51)*⁣**	2.99(2,54)†	53.41(5,51)*⁣**	57.39(5,51)*⁣**

**N* = 57. Effects are unstandardized regression coefficient. Standard errors are in parentheses. ^†^*p* < 0.10; ^∗^*p* < 0.05; ^∗∗∗^*p* < 0.001 (two-tailed).*

**FIGURE 6:**
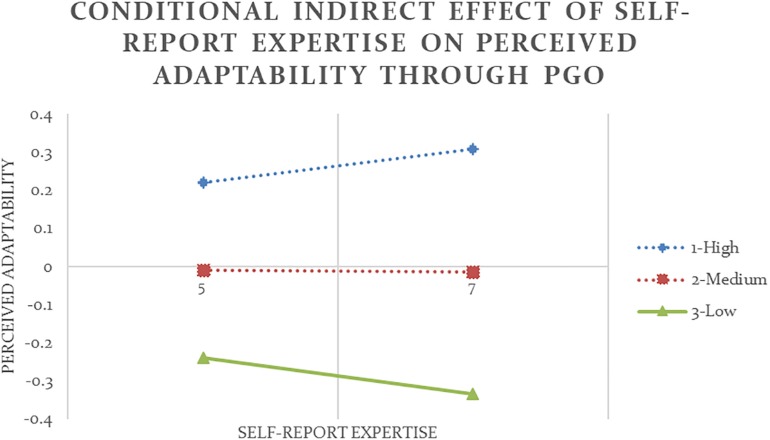
Conditional indirect effect of self-report expertise on other-report adaptability through PGO is significant at low values of the moderator other-report humility with 90% confidence (Analysis #17).

**FIGURE 7:**
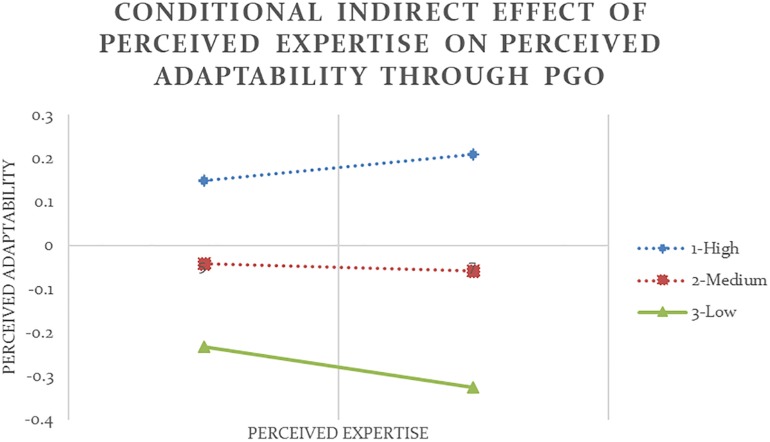
Conditional indirect effect of other-report expertise on other-report adaptability through PGO is significant at low values of the moderator other-report humility with 90% confidence (Analysis #18).

Consistent with previous results, Analysis #17 (see Table 5 and Figure 6) and Analysis #18 (see Table 5 and Figure 7) also showed that other-report humility moderated the indirect effect of expertise, whether self-report or other-report, on other-report adaptability through PGO. Among people who were perceived to have low level of humility, higher expertise led to stronger PGO but stronger PGO led to lower other-report adaptability.

## Discussion

In this study, I used data collected from a small sample of healthcare professionals to test three hypotheses, that PGO would mediate the relationship between expertise and flexibility (*H*_1_), and that LGO (*H*_2_) and humility (*H*_3_) would moderate this indirect effect. All three hypotheses were partially supported.

Overall, results indicated that PGO partially explained the mechanism of why experts may become inflexible, though the mechanism depended on whether the outcome was measured by self-report or other-report. When outcome was measured as self-report, results were in the hypothesized direction, meaning higher self-report expertise led to stronger PGO, which then led to less flexibility. On the other hand, when outcome was measured as other-report, stronger PGO was associated with higher other-report adaptability, making the indirect effect of expertise on flexibility positive instead of negative. This may be logical because external raters may have perceived signs of strong PGO (such as striving to do the work well and avoiding showing weaknesses or failures) as equivalent to adapting in order to achieve success.

Of the two moderators, humility, both as self-report and other-report measures, was the more consistent moderator of the indirect effect of expertise on flexibility through PGO. For those with average or below average levels of self-report humility, higher self-report expertise was associated with lower self-report adaptability. On the other hand, for those with average or above average levels of humility, higher self-report expertise was associated with higher other-report adaptability. Similarly, for those who reported high level of humility, higher other-report expertise was associated with higher other-report adaptability. When experts were perceived to have low levels of humility, higher expertise, whether self-report or other-report, lead to lower other-report adaptability. Taken all together, these results suggested that having low levels of humility added to the pressure to perform to make experts inflexible, while having above average level of humility actually made experts more flexible. This meant that experts who were humbler were less susceptible to the negative effect of PGO and were more skillful in avoiding the inflexibility trap, while their less humble counterparts might have fallen right into it.

LGO only moderated the indirect effect of expertise on flexibility through PGO when both expertise and flexibility were self-report, and the direction of effect was as hypothesized. Among experts who reported average or below average levels of LGO, higher expertise led to higher PGO, which then led to lower adaptability. This implied that if experts only focused on performance and did not spend time to learn and improve their skills, they would have been more likely to suffer from inflexibility, even though putting stronger emphasis on learning might not have brought apparent advantages.

The biggest takeaway from this study is that humility may be a key factor distinguishing inflexible experts from flexible ones. Even though the benefits of humility may not always be apparent, having low (below average) levels of humility can bring serious disadvantages to experts, rendering them incapable of escaping the shadow of their own expertise. Knowing this, managers should consider incorporating humility as a core value of organizations to start building an organizational culture that encourages seeing things in perspectives, seeking feedback and new insights, and continually improving one’s skills.

The small sample size is a major limitation of this study. However, strong power of more than 0.99 as mentioned in the Methods section justified the use of 90% confidence intervals in the study. This study should be treated as an exploratory study—in fact, the first of its kinds—looking into factors that may distinguish flexible experts from inflexible ones. I employed a couple of *a priori* procedural remedies to control for method biases, including obtaining measures of predictor and criterion variables from different sources and eliminating common scale properties by using different types of measurement scales (Podsakoff et al., 2012).

This study contributes to the literature in a number of areas. First, I join the conversation about experts’ inflexibility and propose a motivational framework to explain the same phenomena in a different way. More importantly, my theoretical framework—built on Dweck’s work on lay theories of abilities and goal orientation (Dweck, 1986, 1999, Dweck et al., 1995)—explains why some experts are more flexible than others and offers testable hypotheses and more accessible solutions to the problems of expert performance pitfalls beyond what has been proposed cognitively and behaviorally. Knowing the mechanism causing this problem—the performance pressure in knowledge enterprises—helps future research identify more ways in which this mechanism can be attenuated. This finding is in line with Argyris (1985, 1986, 1991, 1994) observation that the performance evaluation systems at companies are the main culprits of the doom loop, making smart people fall victims to defensive routines and fail to learn and change.

Second, this study is also among the first to test the differentiated roles of LGO and PGO as predictors and moderators of an outcome. Previous research has frequently distinguished and juxtaposed the effects that these two goal orientations have on outcomes such as student GPA (Button et al., 1996), task performance (Davis, 2005), employee creativity (Hirst et al., 2009), or self-efficacy (see Cellar et al., 2011 for a meta-analysis). While this approach has its merits, doing so further reinforced the view that PGO and LGO are diametrically opposite constructs—one that has been stated and confirmed as misleading and incorrect (Dweck, 2006, 2015). Simultaneously examining how one’s PGO and LGO may play different functions in one’s cognitive capacities opens up new possibilities in goal orientation research. In this particular case—to borrow the terminologies from complex systems science—PGO creates a self-limiting feedback loop that limits experts’ flexibility; whereas LGO created a self-reinforcing feedback loop that loosens the first self-limiting loop. Similar to James March’s seminal proposal that “maintaining an appropriate balance between exploration and exploitation is a primary factor in system survival and prosperity” (March, 1991, p. 71), it is likely that experts need a balance of PGO and LGO in order to both maintain their superior performance and adapt to unforeseen circumstances. Future research could also explore how PGO and LGO interact with each other to shape and influence a variety of individual behaviors such as generation and implementation of new ideas, entrepreneurial venturing, lifelong learning and achievement, etc.

Last but not least, I extend the conversation about the importance of humility in today’s organizations and apply it outside of the leader-follower context. I join others in promoting that humility is a virtue that should be valued instead of suppressed. Having humility helps experts be more flexible, while not having it or having below average levels of humility further reinforced the inflexibility trap of expertise. Recent works on humility share some similarities with this finding. In a series of four studies, Porter and Schumann (2018) showed that participants with higher levels of humility were more open to learning about the opposition’s view during disagreements, more likely to expose themselves to opposing political perspectives, and more open to opposing views in general. Weidman et al. (2018) revealed that when humble people celebrated personal success, they also tended to appreciate others’ contribution, display authentic pride, and prestige-based status.

This study hopes to generate awareness for leadership and management teams as well as experts in organizations by showing the importance of humility and learning in sustaining high, adaptive performance. At the individual level, allocating time to update their skills and perform challenging tasks, as well as developing a sense of humility and being aware of their limitations will help expert be more flexible and thus more effective at work. As Schein (2013) noted, in a performance oriented culture that values doing and telling more than asking and relating, people do not spend enough time to learn about others’ interests or concerns before bombarding them with self-righteous information. Instead, they often assume that they know what others want, or what is good for others, and rarely test these assumptions (Buckner and Carroll, 2007). Worthington and Allison (2018) echoed these observations, asserting that it is not easy to develop or practice humility in an individualistic culture. Instead, a great deal of courage, leadership, and heroic self-sacrifice is required to do so. At the very beginning, humility could develop “from having secure attachment relationships and the ability to bounce back from adversity” (Dwiwardani et al., 2014, p. 83). Empathizing with others’ experience and rediscovering the experience of being inexperienced (Zhang, 2015) could also help experts become more humble.

On a strategic level, in order to foster an organizational culture that is open to learning and receptive of humility, leaders and managers can help by demoting the image of experts as all-knowing and always correct, encouraging risk-taking, making it acceptable to make mistakes, building time for feedback and learning into project tasks, and developing interdepartmental collaborations. This kind of environment will help experts engage in more double-loop learning (Argyris, 1994) instead of avoiding learning-provoking conversations (Argyris, 1986). It is also crucial that leaders themselves practice humility and model this behavior (Owens and Hekman, 2012) to create a climate that is psychologically safe for others to do the same (Edmondson, 1999), and eventually help preventing organizational defensive routines (Argyris, 1985). Recent research has found that leader humility is positively related to employees’ perspective taking and creativity (Wang et al., 2017), follower performance (Diao et al., 2019), and employee voice (Lin et al., 2019). Humble CEOs tend to gather a top management team that is more likely to collaborate, have a shared vision, and share information as well as the decision-making process (Ou et al., 2018). The opposites of these—overconfidence, secretive and biased communication, unrealistic performance pressure, widespread fear, and inhibited innovation—were how Nokia lost the smart phone battle (Vuori and Huy, 2016).

## Conclusion

Acquiring and training experts to be able to quickly respond to the changing environments has been and will always be a big challenge for all organizations in this day and age. The solution sometimes may be counterintuitive: that one has to unlearn what one has learned, refrain from doing what has been successful, and keep in perspective what one has achieved. Having a piece of the humble pie and the mindset to continually learn new practices will help our knowledge experts go farther and be more resilient in today’s uncertain world.

## Data Availability Statement

The data analyzed in this manuscript will be made available by the author to any qualified researcher upon request.

## Ethics Statement

This study was carried out in accordance with the recommendations of Case Western Reserve University IRB with written informed consent from all subjects. All subjects gave written informed consent in accordance with the Declaration of Helsinki. The protocol was approved by the Case Western Reserve University IRB.

## Author Contributions

MT: conceptualization, methodology, investigation, formal analysis, resources, data curation, writing (original draft), visualization, supervision, and project administration.

## Conflict of Interest

The authors declare that the research was conducted in the absence of any commercial or financial relationships that could be construed as a potential conflict of interest.
